# On the de­pro­ton­ation of chloro­thia­zide

**DOI:** 10.1107/S2053229625000701

**Published:** 2025-01-30

**Authors:** Rowan K. H. Brydson, Morven L. Gray, Alan R. Kennedy, Benjamin C. O’Hara, Michael W. Reid, Ifeka Ugbolue

**Affiliations:** a Department of Pure & Applied Chemistry, University of Strathclyde, 295 Cathedral Street, Glasgow G1 1XL, Scotland, United Kingdom; Hong Kong University of Science and Technology, Hong Kong

**Keywords:** crystal structure, pharmaceuticals, salt selection, sulfonamide, alkali metals, diuretic

## Abstract

Three alkali metal salt forms of the diuretic chloro­thia­zide (systematic name: 6-chloro-1,1-dioxo-2*H*-1,2,4-benzo­thia­zine-7-sulfonamide, HCTZ) are described. When crystallized from aqueous solution, the Na and K salts, namely, poly[[μ-aqua-aqua­(μ_3_-6-chloro-1,1-dioxo-7-sulfamoyl-2*H*-1,2,4-benzo­thia­diazin-2-ido)sodium] hemihydrate], {[Na(C_7_H_5_ClN_3_O_4_S_2_)(H_2_O)_2_]·0.5H_2_O}_*n*_, and poly[[di­aqua­(μ_5_-6-chloro-1,1-dioxo-7-sulfamoyl-2*H*-1,2,4-benzo­thia­diazin-2-ido)potassium] hemihydrate], {[K(C_7_H_5_ClN_3_O_4_S_2_)(H_2_O)_2_]·0.5H_2_O}_*n*_, are both found to have stoichiometry *M*CTZ·2.5H_2_O, with CTZ de­pro­ton­ated at a heterocyclic ring N atom. Both the stoichiometry and the de­pro­ton­ation site are different to those described in previously published versions of these structures. The Cs salt form is found to be the monohydrate CsCTZ·H_2_O, namely, poly[[aqua­(μ_5_-6-chloro-1,1-dioxo-7-sulfamoyl-2*H*-1,2,4-benzo­thia­diazin-2-ido)caesium], [Cs(C_7_H_5_ClN_3_O_4_S_2_)(H_2_O)]_*n*_. As with the Na and K cognates, this structure is also de­pro­ton­ated at the heterocyclic ring. NaCTZ is found to be a two-dimensional coordination polymer with bridges between Na centres formed by H_2_O and SO_2_ groups, and by links through the length of the coordinated CTZ anions. Water ligands in KCTZ and CsCTZ are terminal, rather than bridging between metal centres, but both com­pounds form structures where *M*—Cl inter­actions link two-dimensional motifs formed *via M*—O bonds (and in CsCTZ, *M*—N bonds) into three-dimensional coordination polymers.

## Abstract 2

The structures of the Na and K salt forms of chloro­thia­zide have been re-investigated and are found to have different hydration states and de­pro­ton­ation sites from those described previously. The structure of the Cs salt form is found to contain the same anion as in the new models for the Na and K salt forms.

## Introduction

The active pharmaceutical ingredient (API) chloro­thia­zide and its sodium salt (NaCTZ, where CTZ is the 6-chloro-1,1-dioxo-7-sulfamoyl-2*H*-1,2,4-benzo­thia­diazin-2-ide anion) are sulfonamide com­pounds utilized as diuretic and anti­hy­per­ten­sive drugs (Martins *et al.*, 2022 ▸; Steuber *et al.*, 2020 ▸). Chloro­­thia­zide has also been widely used as a model API in crystallization studies. These studies have identified two poly­morphs under ambient conditions and an additional high-pressure polymorphic form of chloro­thia­zide (Shankland *et al.*, 1997 ▸; Brydson & Kennedy, 2024 ▸; Oswald *et al.*, 2010 ▸), as well as numerous solvate and cocrystal forms (*e.g.* Johnston *et al.*, 2011 ▸; Aljohani *et al.*, 2017 ▸; Teng *et al.*, 2020 ▸). Despite this widespread study, and despite NaCTZ being used as an injectable form of the drug (Hankins *et al.*, 2001 ▸), only four structures of salt forms of chloro­thia­zide have been reported. These are APUZER [Cambridge Structural Database (CSD, Version 5.45 with updates to June 2024) refcode; Groom *et al.*, 2016 ▸], which was reported as a trihydrate form of NaCTZ (Paluch *et al.*, 2010 ▸), APUZIV and APUZOB which were, respectively, reported as the dihydrate and the mixed hydrate/ethano­late forms of KCTZ (Paluch *et al.*, 2011 ▸), and VEKBOF, which has the organic cation PhC(NH_2_)_2_ (Aljohani *et al.*, 2017 ▸). The alkali metal salt forms are of particular pharmaceutical inter­est, as they are reported to have aqueous solubilities that are orders of magnitude greater than that of chloro­thia­zide itself (Paluch *et al.*, 2010 ▸, 2011 ▸).
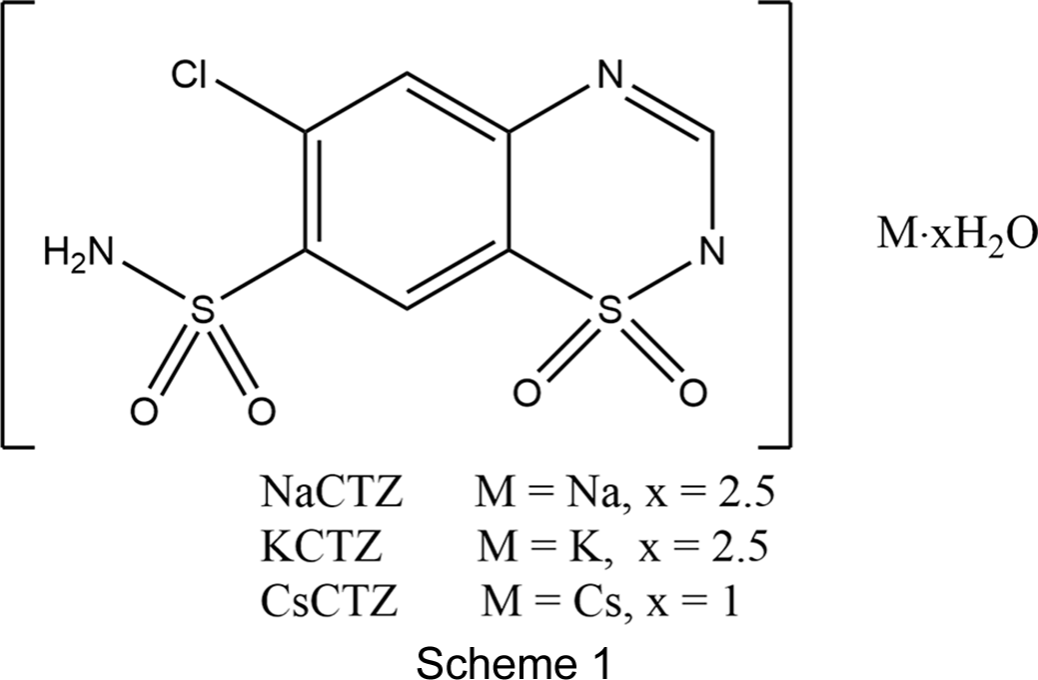


Our attention was originally drawn to APUZER as its two-dimensional diagram in the CSD features a neutral CTZ ligand with the charge on Na^+^ being balanced by a hydroxide ligand. Examining the associated CIF and the original article quickly showed that this was a transcription error (Paluch *et al.*, 2010 ▸). However, the anionic form of CTZ that is reported is in itself unusual. The structure given shows de­pro­ton­ation of the SO_2_NH_2_ group and a proton present on the thia­diazine ring N atom adjacent to the ring SO_2_ group. This is unusual as the SO_2_NH_2_ group should be less acidic that the ring N—H group, and the solid-state structures of neutral CTZ forms invariably report the tautomer with the heterocyclic ring protonated at the N atom *para* to the SO_2_ functionality (*e.g.* Brydson & Kennedy, 2024 ▸; Johnston *et al.*, 2011 ▸; Aljohani *et al.*, 2017 ▸). The KCTZ salt forms APUZIV and APUZOB are reported to have the same de­pro­ton­ation pattern as APUZER (Paluch *et al.*, 2011 ▸), but the organic salt VEKBOF has a CTZ anion with the more intuitive de­pro­ton­ation of the N—H group of the heterocyclic ring and retention of the SO_2_NH_2_ group (Aljohani *et al.*, 2017 ▸).

It is noted that the H-atom modelling in the reported <!?tlsb=-0.08pt>structures of all three alkali metal salt forms has some problems. Notably, some H atoms on water mol­ecules are missing, some refined N—H distances are unreasonably short (*e.g.* 0.65 Å), and those O—H and N—H bond lengths that are reasonable have all been fixed at the distances given. As incorrect H-atom positions are a known pitfall even for relatively high-quality crystal structure determinations (Seidel, 2018 ▸; Kennedy *et al.*, 2023 ▸; Bernal & Watkins, 2013 ▸; Raymond & Girolami, 2023 ▸; Harlow, 1996 ▸), we investigated the de­pro­ton­ation of CTZ by redetermining the structures of the hydrated NaCTZ and KCTZ forms and by determining a related new structure – that of a monohydrated form of CsCTZ.

## Experimental

### Synthesis and crystallization

The triclinic polymorph of CTZ was purchased from Thermo Scientific. Crystals of NaCTZ were prepared ac­cord­ing to the aqueous method of Paluch *et al.* (2010 ▸). Crystals of KCTZ were prepared by adding excess KCl to an aqueous solution of NaCTZ, followed by slow evaporation of the solvent. For the preparation of CsCTZ, CTZ (0.10 g, 0.34 mmol) was dissolved in the minimum amount of a 1:1 (*v*/*v*) acetone–water mix. To this was added CsOH·H_2_O (0.06 g, 0.36 mmol) dissolved in the minimum amount of water. After stirring and heating, the resulting solution was left to evaporate for 3 d at room temperature. This gave crystals of CsCTZ in approximately 50% yield. FT–IR (cm^−1^); 3422, 3308, 3258, 3082, 2959, 1602, 1573, 1509, 1466, 1300, 1246, 1152, 1094, 956, 893, 714, 674, 614, 524.

### Refinement

Crystal data, data collection and structure refinement details are summarized in Table 1 ▸. All H atoms were observed by difference synthesis, except for those of the disordered water mol­ecule of NaCTZ. The H atoms of this latter group were thus placed in positions calculated so as to give sensible inter­molecular hy­dro­gen-bonding inter­actions. H atoms bound to C atoms were placed in expected geometric positions and treated in riding modes, with C—H = 0.95 Å and *U*_iso_(H) = 1.2*U*_eq_(C). Well-ordered H atoms bound to N or to O atoms were placed as found and refined isotropically with N/O—H distances restrained to 0.88 (1) Å.

## Results and discussion

**Figure 1:**
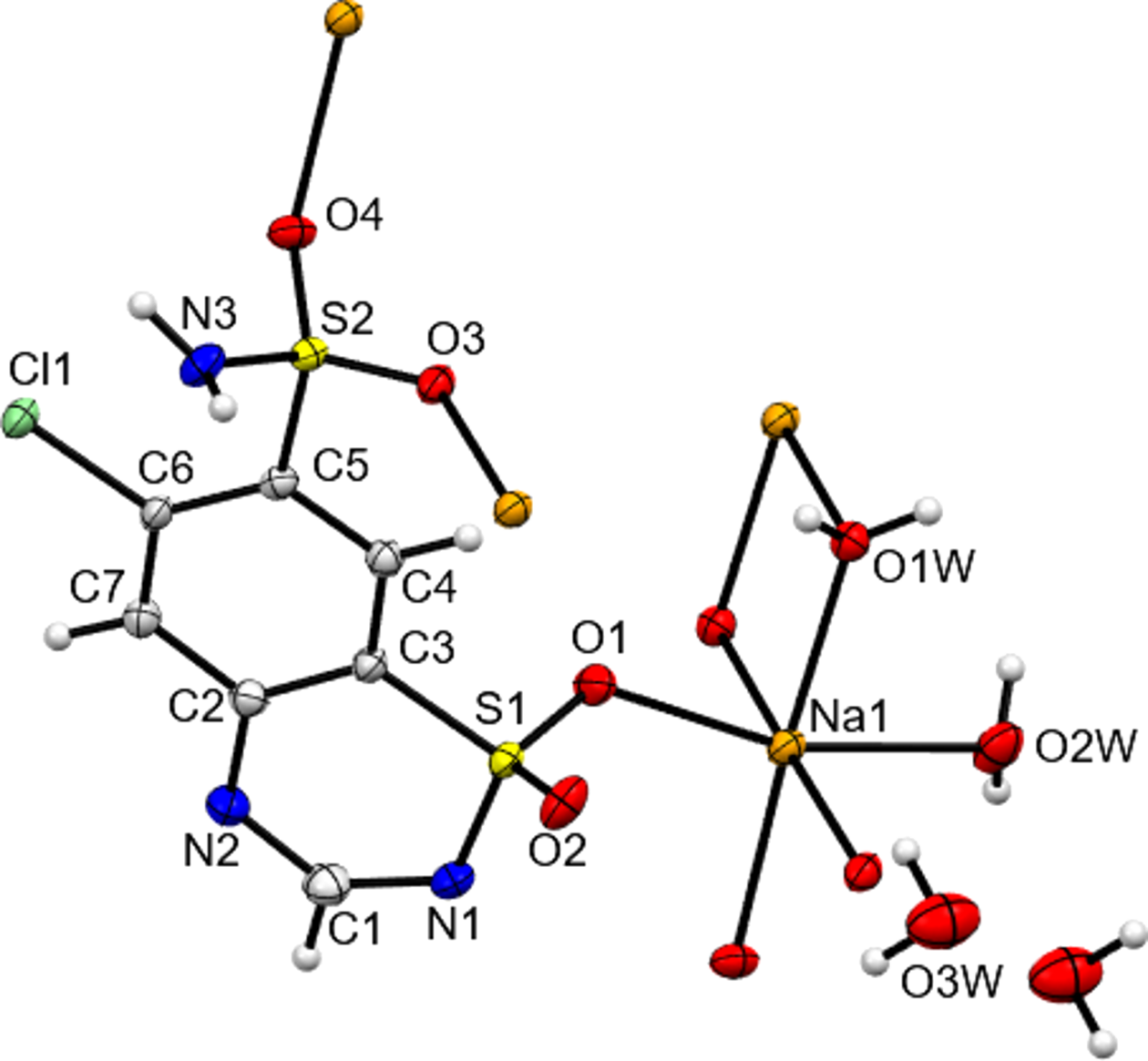
Contents of the asymmetric unit of NaCTZ, expanded so as to show all metal-to-ligand coordination bonds. Note that here and elsewhere, non-H atoms are drawn as 50% probability ellipsoids and H atoms as small spheres of arbitrary size. See supporting information for full details of bonding contacts, including symmetry operations, for all structures.

The core structures of NaCTZ, KCTZ and CsCTZ as newly determined herein are shown in Figs. 1 ▸–3 ▸ ▸ and key crystallographic parameters are given in Table 1 ▸. Paluch *et al.* (2010 ▸) modelled the structure of NaCTZ in APUZER (CSD refcode) as a trihydrate, with two water ligands coordinated to sodium and one free water mol­ecule ‘of solvation’. Both in the original article and in our hands, using this model gives the free water mol­ecule an extremely large displacement ellipsoid and results in an O⋯O separation of just 1.482 Å between two free water mol­ecule sites related by a centre of symmetry. In our current model, we thus treat this site, O3*W*, as a half-occupancy water mol­ecule. This gives normal displacement ellipsoids, removes the erroneous O⋯O separation and re­inter­prets the structure as NaCTZ·2.5H_2_O. In the text of Paluch *et al.* (2011 ▸), KCTZ (APUZIV) is described as a dihydrate form. However, both the CIF file deposited for APUZIV and our redetermination show that, similar to the Na salt, the K salt has stoichiometry KCTZ·2.5H_2_O. Note that for both NaCTZ and KCTZ, a water content of 2.5 water mol­ecules per cation is closer to the reported TGA derived water contents than are the alternative descriptions of these structures (Paluch *et al.*, 2010 ▸, 2011 ▸).

In both the original structures of NaCTZ and KCTZ (APUZER and APUZIV), some water H atoms were omitted, a H atom was placed on a heterocyclic N atom and the pendant arm was modelled as the de­pro­ton­ated SO_2_NH group (Paluch *et al.*, 2010 ▸, 2011 ▸). In the current work, all the H atoms were observed in difference syntheses maps, with the exceptions of the H atoms of the disordered half-occupancy water mol­ecule of NaCTZ. Adding the H atoms in the observed positions and modelling freely and isotropically gave structurally sensible H-atom positions for the water mol­ecules and gave CTZ anions that had intact SO_2_NH_2_ groups and no protons on the heterocyclic N atoms. Moreover, there were no electron-density features suggesting any degree of protonation of the heterocyclic N atoms. Difference electron-density maps for NaCTZ and KCTZ are available as supporting information. As some O—H distances of the freely refined models were slightly short (0.79 Å), the final reported models restrained *X*—H (*X* = O or N) to be 0.88 (1) Å (see Tables 2 ▸–4 ▸ ▸). Similar treatment of CsCTZ gave a structure with the same protonation behaviour for the CTZ anion as was found herein for NaCTZ and KCTZ. The H atoms of the disordered half-occupancy water mol­ecule of NaCTZ were added in calculated positions that gave sensible inter­molecular hy­dro­gen-bonding contacts (see Table 2 ▸). Thus, electron-density data clearly gives models for both NaCTZ and KCTZ that differ from those reported as APUZER and APUZIV. We think it is clear that these structures should have been described as having intact SO_2_NH_2_ groups and as having been de­pro­ton­ated at the heterocyclic ring.

The K salt APUZOB is a mixed ethano­late/hydrate that was also reported to have a de­pro­ton­ated SO_2_NH unit (Paluch *et al.*, 2011 ▸). We were unable to obtain crystals of this form, but have investigated its de­pro­ton­ation site by com­paring the various bond lengths involving the N atoms of the CTZ anions. Study of Table 5 ▸ shows clear geometric differences between the salt forms that contain de­pro­ton­ated CTZ anions and the neutral polymorphs of chloro­thia­zide (Leech *et al.*, 2008 ▸; Brydson & Kennedy, 2024 ▸). All the salt forms show similar bond lengths to each other, including the two crystallographically independent CTZ anions of APUZOB. We believe that as this group forms a coherent set, it indicates that APUZOB may also have been incorrectly reported with respect to the de­pro­ton­ation site, and that it should also be de­pro­ton­ated at the ring N atom. Note that in the neutral polymorphs the C1—N1 bond is considerably shorter than the C2—N1 bond, indicating that it is mostly C1—N1 that has double-bond character. In contrast, for the anionic CTZ forms, C1—N1 is slightly longer than C2—N1. The chemical scheme has been drawn so as to place the double bond at the shorter C2—N1 site, but of course such small differences mean that in reality an inter­mediate resonance form is observed.

**Figure 4:**
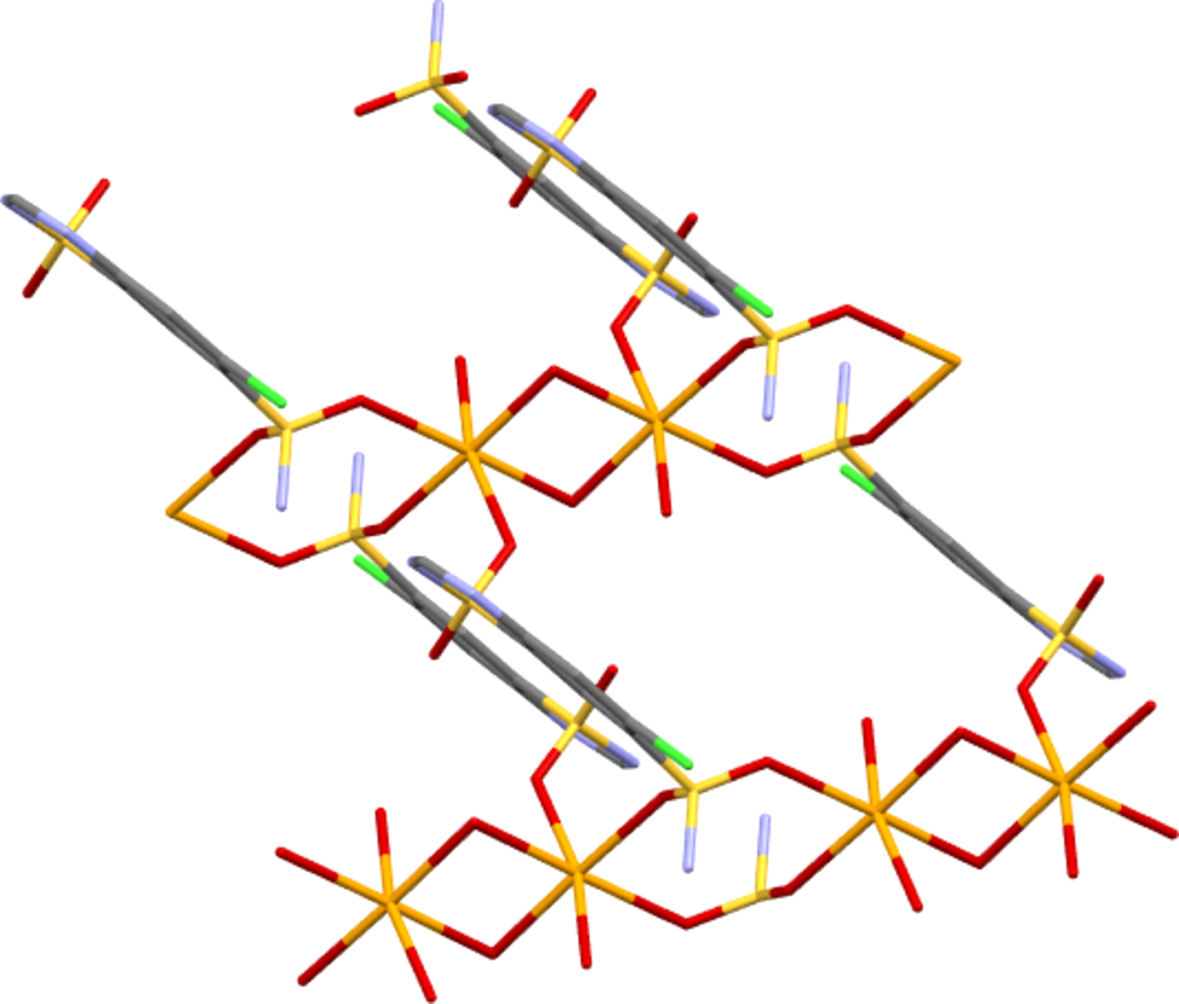
Detail of the coordination bonding in NaCTZ, showing one-dimensional chains with [NaONaO] and [NaOSO]_2_ rings linked into a two-dimensional motif by CTZ anions bridging between the chains.

**Figure 5:**
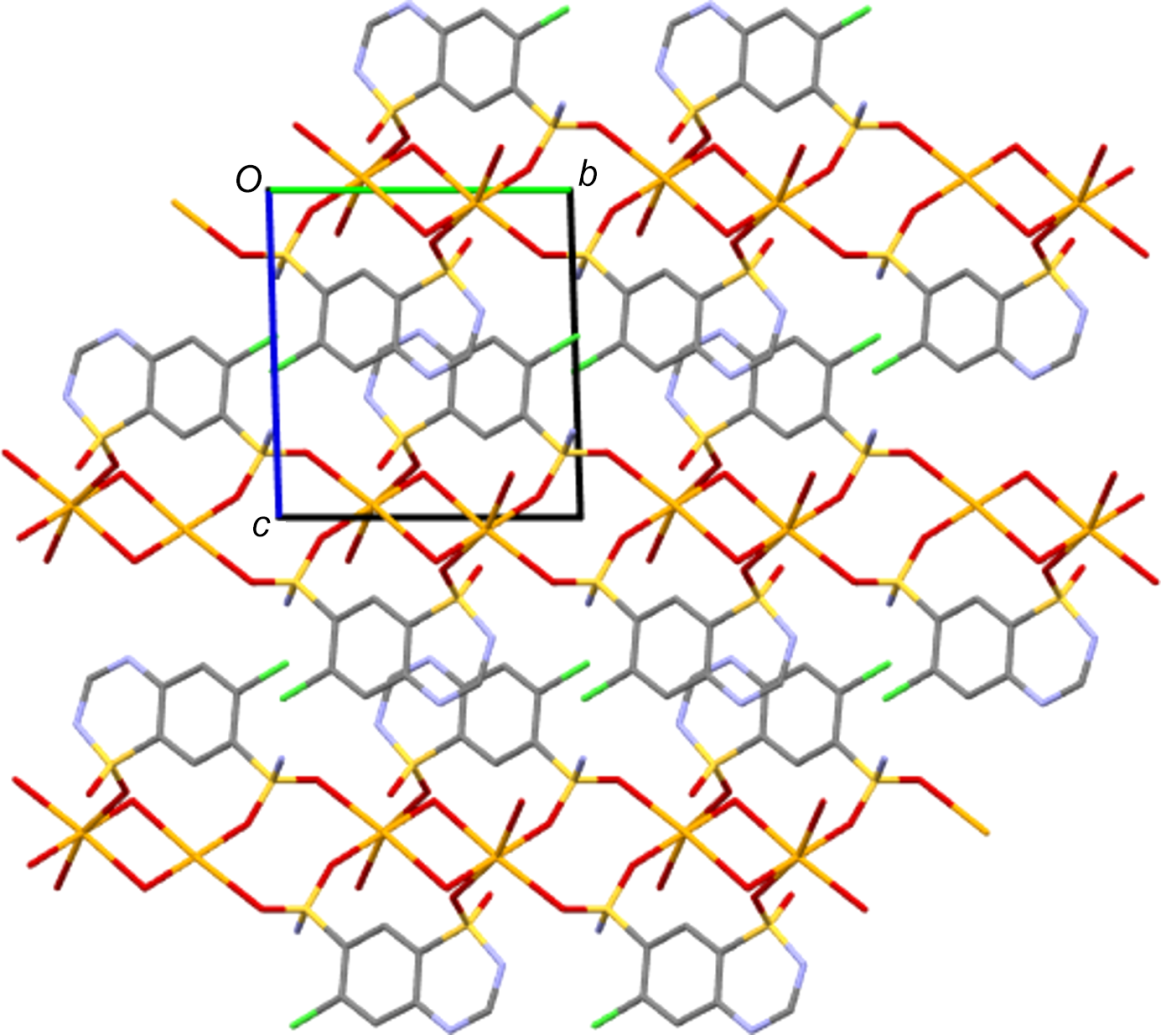
Packing structure of NaCTZ, viewed along the *a* axis and showing organic and inorganic layers alternating along the *c* direction.

NaCTZ has a six-coordinate octa­hedral Na centre with an O_6_ coordination set. Three of these O atoms are from water ligands (two bridging between Na centres and one terminal) and the other three are from SO_2_ units of the CTZ anion. Details of coordination bonds for the three salt forms are given in Table 6 ▸. All three of these SO_2_—Na bonds lead to bridges between Na centres. The SO_2_NH_2_ unit forms eight-membered [NaOSO]_2_ rings which alternate with four-membered NaONaO rings (O from water) to propagate the structure perpendicular to the crystallographic *b* direction. The CTZ anions bridge between these chains *via* Na bonds to both SO_2_ groups of CTZ to give connectivity parallel to the crystallographic *a* direction, giving an overall two-dimensional co­ordination polymer (see Fig. 4 ▸). Hydro­philic inorganic layers thus alternate with hydro­phobic organic bilayers along the *c* direction, with the main CTZ-to-CTZ inter­actions across the organic bilayers being from N—H⋯N hy­dro­gen bonds (Table 2 ▸ and Fig. 5 ▸).

**Figure 6:**
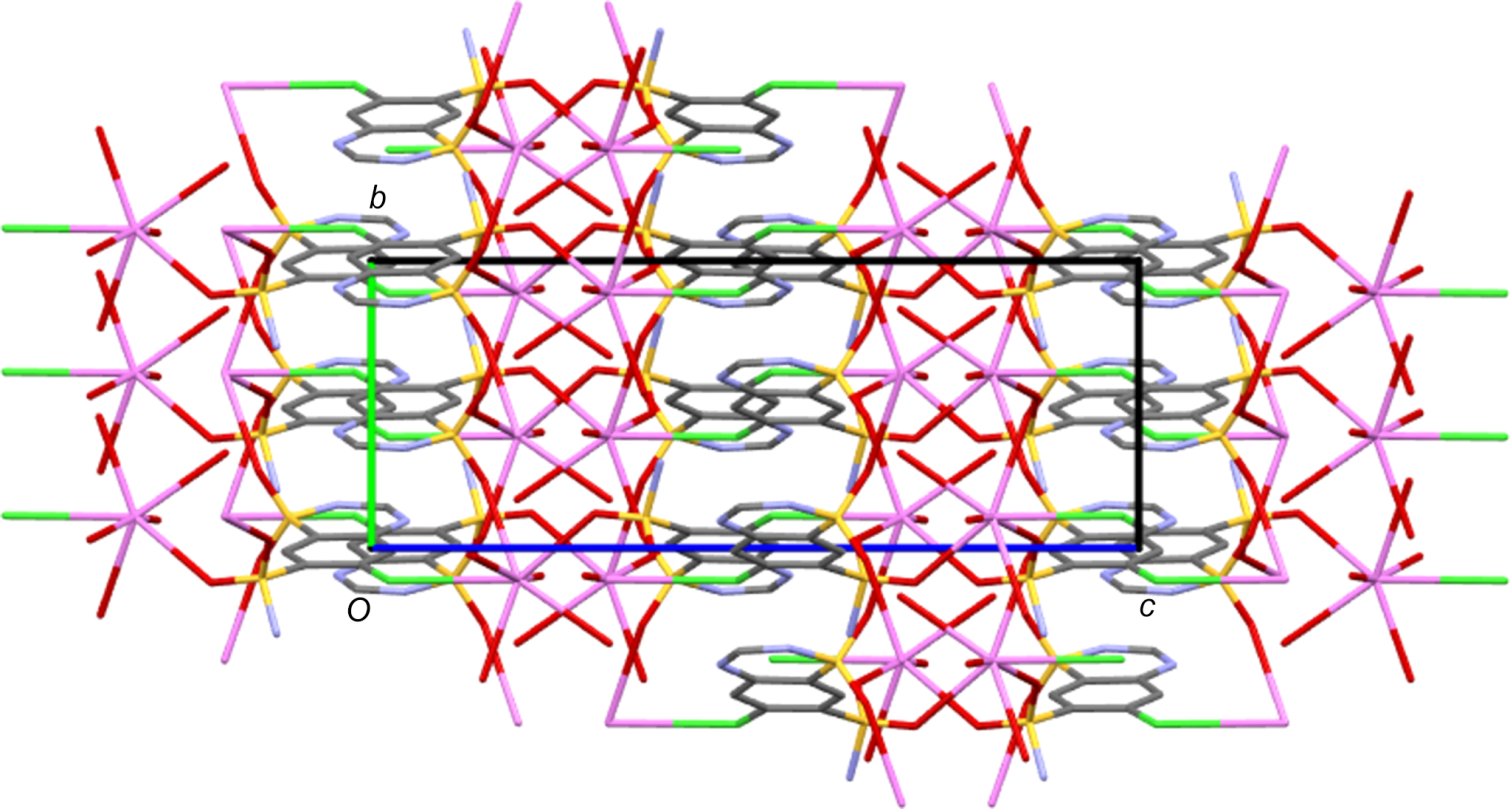
Packing structure of KCTZ, viewed along the *a* axis and showing organic and inorganic layers alternating along the *c* direction.

The K centre in KCTZ is seven-coordinate and has a somewhat unusual O_6_Cl coordination shell. The two water ligands are terminal and thus the coordination polymer builds solely through inter­actions with the CTZ anions. The unbound water mol­ecule sits on a crystallographic twofold axis, giving an overall stoichiometry of KCTZ·2.5H_2_O. Each K centre bonds to four CTZ anions through inter­actions with all four chemically distinct O atoms of CTZ. There is also a relatively unusual bond to Cl of a CTZ anion. At 3.3257 (4) Å, the K—Cl bond with the organic halide is similar to, or only slightly longer than, typical bond lengths reported between K and chloride anions (*e.g.* 3.325 and 3.094 Å in ZUKDUH and BEPSAS, respectively) (Zaleskaya *et al.*, 2020 ▸; Yang *et al.*, 2013 ▸). Alkali metal to organic halide bonds are described in the literature, but most are observed with simple polyhalogenated aromatics and relatively few with less substituted rings (*e.g.* Smith, 2015 ▸; Rosokha *et al.*, 2009 ▸; Mastropierro *et al.*, 2022 ▸; Osterloh *et al.*, 2001 ▸). A rare example of such a bond in a drug material is the Na—Cl bond observed in the structure of the Na salt of diclofenac (Oyama *et al.*, 2021 ▸). With each K centre making bonds with five neighbouring CTZ anions, the result is a three-dimensional coordination polymer as shown in Fig. 6 ▸. K-to-O inter­actions form a two-dimensional structure parallel to the crystallographic *c* direction and it is the K—Cl bonds that link these layers into the three-dimensional coordination polymer. These bonds in the third di­men­sion are supported by N—H⋯N hy­dro­gen bonds and by hy­dro­gen bonds involving both coordinated and noncoordinated water mol­ecules. The overall packing structure displays inorganic and organic layers alternating along the crystallographic *c* direction (see Fig. 6 ▸).

**Figure 7:**
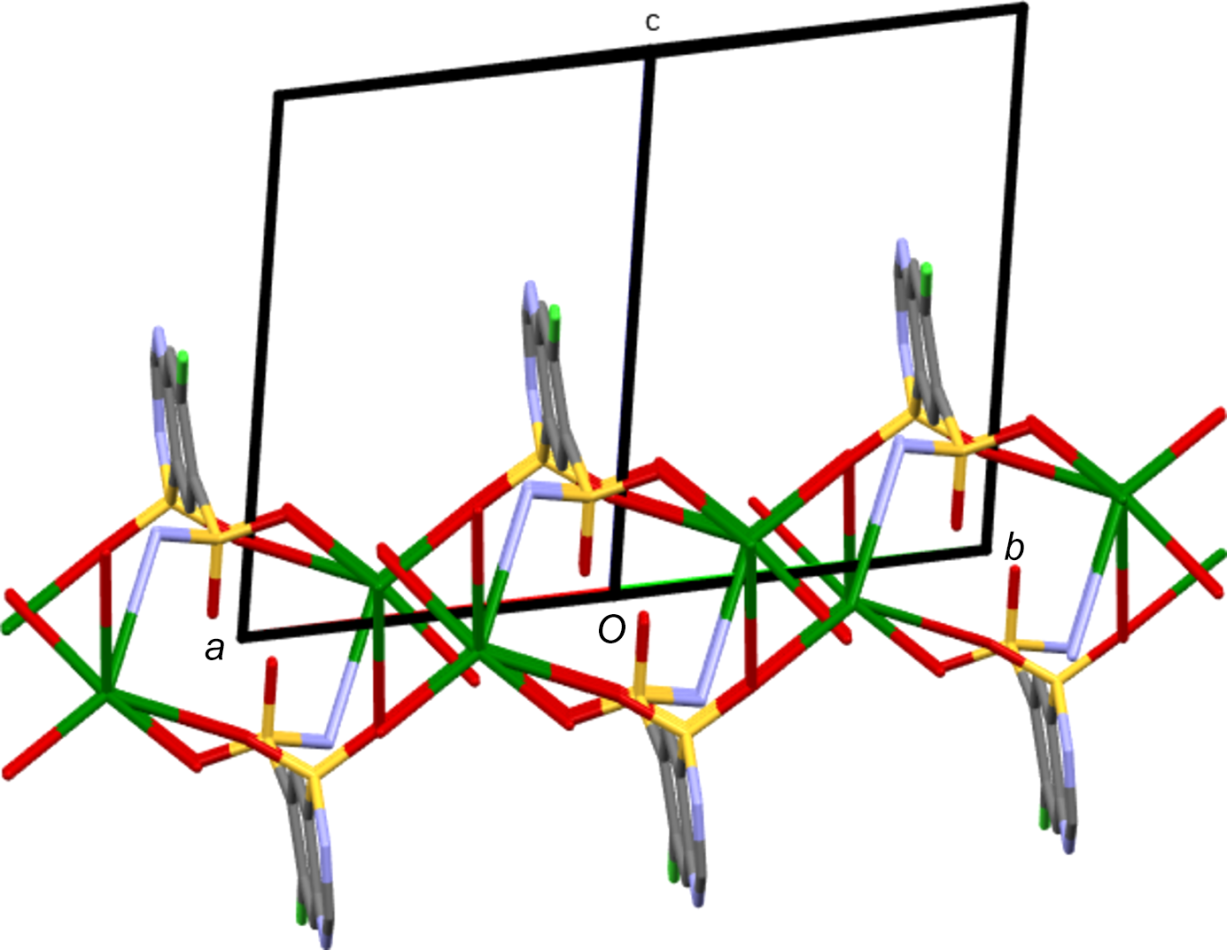
Part of the structure of KCTZ, showing the two-dimensional coordination motif formed by Cs—O and Cs—N bonds. Cs—Cl and hy­dro­gen bonds link neighbouring motifs, along the *c* direction, into a three-dimensional network.

Despite the large size of the Cs cation, CsCTZ has a Cs centre with a maximum of seven dative bonds, the same as found for K in KCTZ. These form an O_5_NCl coordination shell. Although consistent with the treatment of KCTZ above, it is debatable whether or not the Cs—Cl contact of 3.7738 (9) Å should be considered as a dative bond, because although this distance is shorter than the sum of the van der Waals radii for the two atoms, it is longer than the sum of the ionic radii. A search of the CSD showed that the Cs—Cl con­tact herein is approximately 0.2–0.4 Å longer than contacts described as Cs—Cl bonds, but that some structures do include similar distances as formal *R*—Cl bonds to Cl atoms (*e.g.* XELZAQ, NEPNIH and DIQZAG) (Cametti *et al.*, 2006 ▸; Smith, 2013*a* ▸,*b* ▸). CsCTZ is the only structure herein to form an *M*—N bond, and it is notable that this bond is not with a formally charge-carrying ring N atom, but is with the N3 atom of the SO_2_NH_2_ group. At 3.457 (3) Å, the Cs—N bond is considerably longer than the Cs—O bonds [range 2.952 (3)–3.244 (4) Å]. As with KCTZ, the sole water ligand is terminal. The other six inter­actions involve a Cs centre contacting six different neighbouring CTZ anions. The bonds to O and to N give a two-dimensional coordination polymer lying parallel to the crystallographic *ab* plane (see Fig. 7 ▸). Contacts between these planes which would result in a three-dimensional construct are limited to the Cs—Cl inter­actions discussed above and to N—H⋯N hy­dro­gen bonds, with the latter motif being similar to that found in NaCTZ. Again, as in NaCTZ, a layered structure is formed with inorganic layers and organic bilayers alternating along the crystallographic *c* direction.

In all three structures, both ring N atoms act as hy­dro­gen-bond acceptors (see Tables 2 ▸–4 ▸ ▸). The ring sulfonamide N atom always accepts a single hy­dro­gen bond from a neighbouring NH_2_ moiety. The closeness in space of the two N atoms of these inter­actions may go some way to explaining why APUZER and APUZIV incorrectly assign an H atom to the ring rather than to NH_2_. In all three structures, ring atom N2 accepts a hy­dro­gen bond from a metal-coordinated water mol­ecule and, in the case of KCTZ only, it also accepts a second hy­dro­gen bond from a NH_2_ group. As well as the inter­actions described above, the NH_2_ groups of NaCTZ and CsCTZ also donate hy­dro­gen bonds to water mol­ecules. Only in KCTZ does the NH_2_ group act as a hy­dro­gen-bond acceptor, accepting a bond from the non-metal-coordinated water mol­ecule. The O atoms of the SO_2_ groups of the CTZ anions only accept hy­dro­gen bonds from water mol­ecules and thus make no CTZ-to-CTZ contacts.

## Summary

Both modelling electron density and geometric com­parisons with other structures suggest that the previously reported NaCTZ and KCTZ structures APUZER and APUZIV have been misidentified both in terms of hydration state and in terms of the de­pro­ton­ated site of the CTZ anion. Both should have the formula *M*CTZ·2.5H_2_O and both should feature de­pro­ton­ation of the CTZ heterocyclic ring, rather than of the SO_2_NH_2_ group. The Cs salt CsCTZ is found to crystallize as CsCTZ·H_2_O and has the same de­pro­ton­ation site on the CTZ heterocyclic ring as do its Na and K cognates. An unusual feature for salt structures of drug anions is that both KCTZ and CsCTZ display *M*—Cl contacts with the chloro­benzene group, although that in the Cs salt is relatively long. Both these structures give coordination polymers where *M*—O, or *M*—O and *M*—N, bonds give two-dimensional moieties. It is the *M*—Cl contact that expands the K and Cs coordination polymers into the third dimension. Lacking any Na—Cl contact, the structure of NaCTZ remains a two-dimensional coordination polymer.

## Supplementary Material

Crystal structure: contains datablock(s) NaCTZ, KCTZ, CsCTZ, global. DOI: 10.1107/S2053229625000701/qf3065sup1.cif

Structure factors: contains datablock(s) NaCTZ. DOI: 10.1107/S2053229625000701/qf3065NaCTZsup2.hkl

Structure factors: contains datablock(s) KCTZ. DOI: 10.1107/S2053229625000701/qf3065KCTZsup3.hkl

Structure factors: contains datablock(s) CsCTZ. DOI: 10.1107/S2053229625000701/qf3065CsCTZsup4.hkl

CCDC references: 2419938, 2419937, 2419936

## Figures and Tables

**Figure 2:**
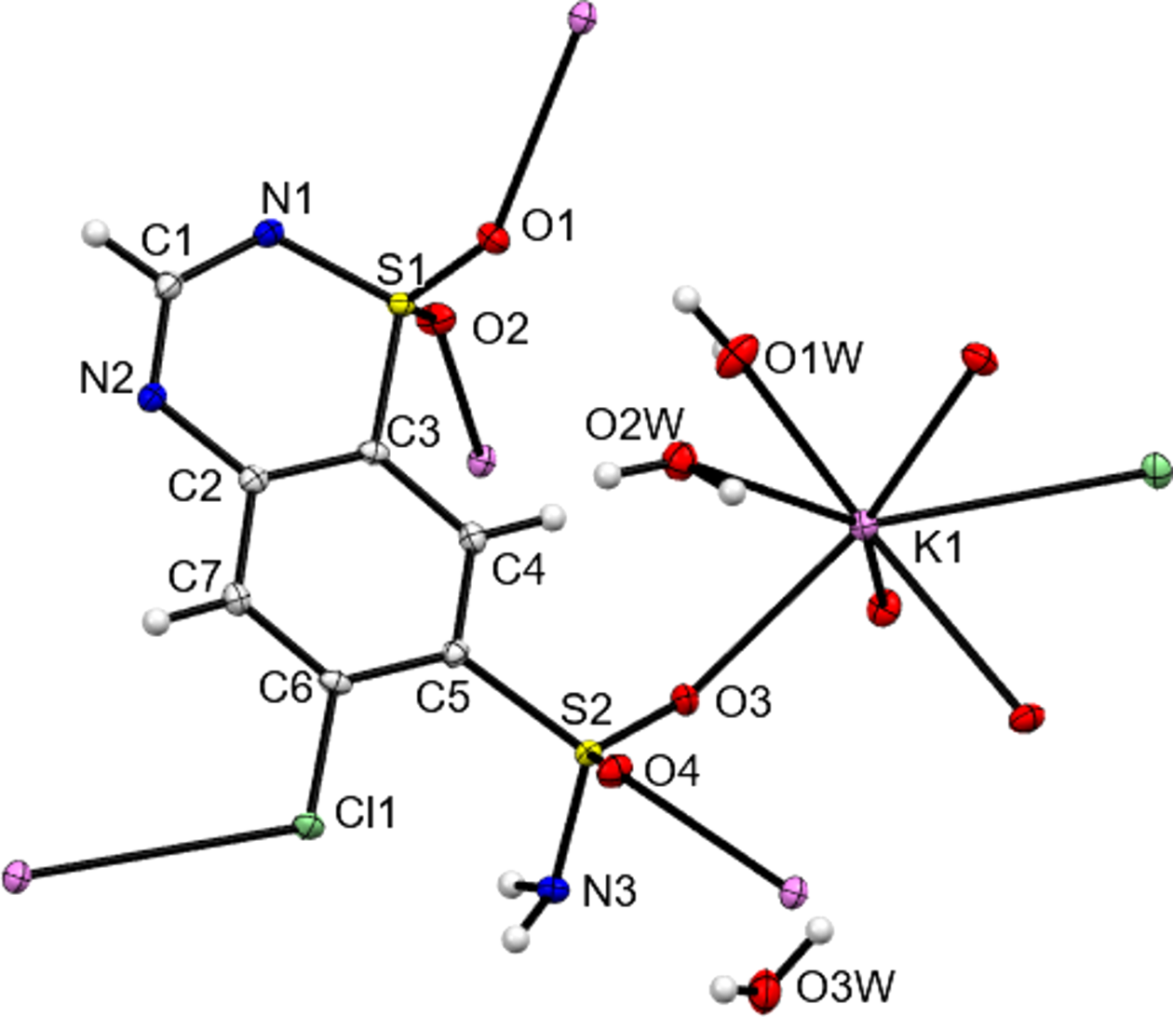
Contents of the asymmetric unit of KCTZ, expanded so as to show all metal-to-ligand coordination bonds.

**Figure 3:**
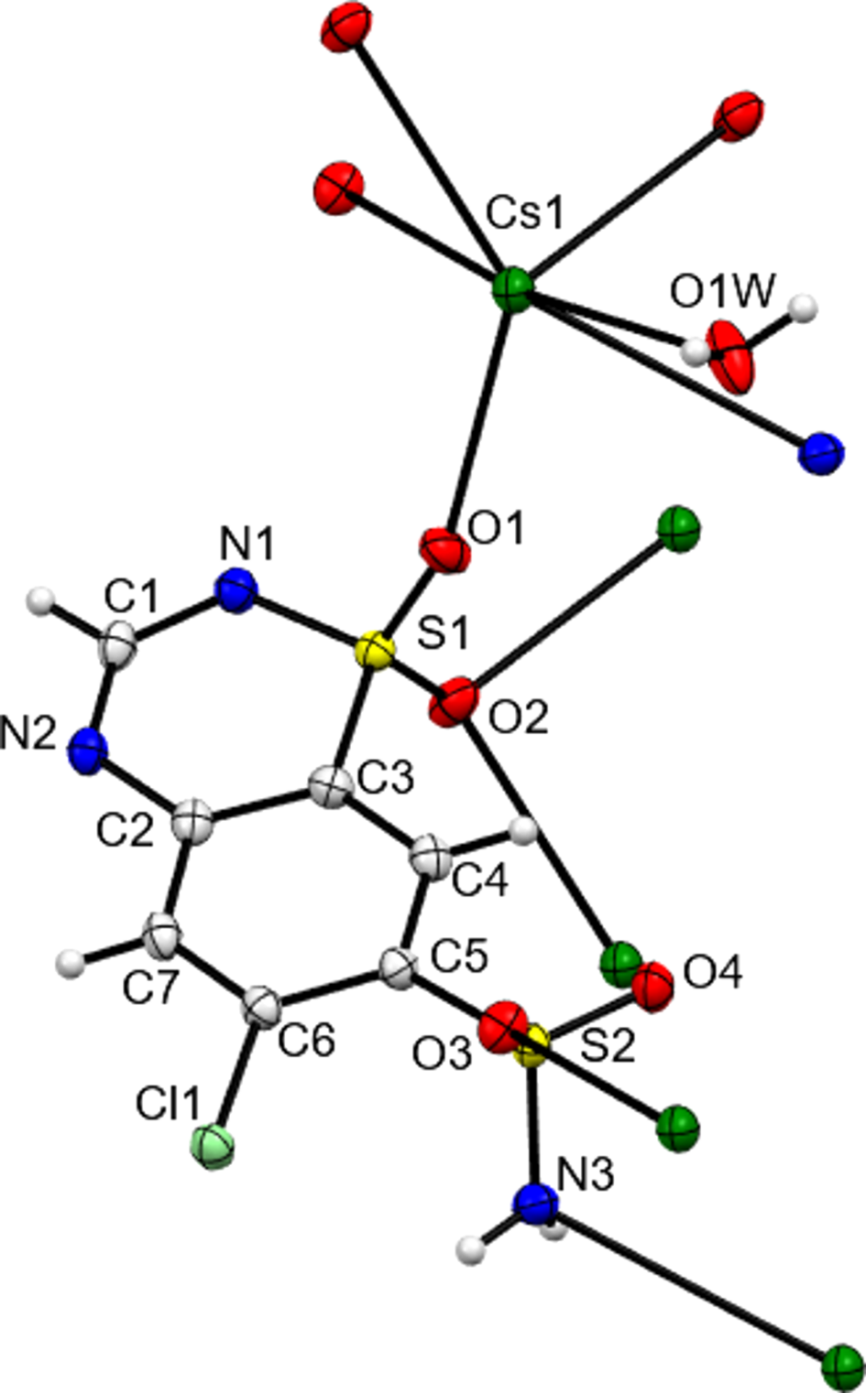
Contents of the asymmetric unit of CsCTZ, expanded so as to show all metal-to-ligand coordination bonds.

**Table 1:** Experimental details Experiments were carried out at 100 K with Cu *K*α radiation using a Rigaku Synergy-i diffractometer. H atoms were treated by a mixture of independent and constrained refinement.

	NaCTZ	KCTZ	CsCTZ
Crystal data			
Chemical formula	[Na(C_7_H_5_ClN_3_O_4_S_2_)(H_2_O)_2_]·0.5H_2_O	[Na(C_7_H_5_ClN_3_O_4_S_2_)(H_2_O)_2_]·0.5H_2_O	[Cs(C_7_H_5_ClN_3_O_4_S_2_)(H_2_O)]
*M*_r_	362.74	378.85	445.64
Crystal system, space group	Triclinic, *P* 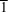	Monoclinic, *C*2/*c*	Triclinic, *P* 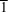
*a*, *b*, *c* (Å)	8.3728 (7), 9.0819 (8), 9.6533 (6)	18.3139 (2), 7.3622 (1), 19.9670 (2)	7.71260 (1), 9.05930 (1), 10.13810 (1)
α, β, γ (°)	83.013 (6), 74.055 (6), 70.189 (7)	90, 99.734 (1), 90	93.9760 (1), 107.5390 (1), 107.8000 (1)
*V* (Å^3^)	663.70 (10)	2653.40 (5)	632.84 (1)
*Z*	2	8	2
μ (mm^−1^)	6.16	8.66	28.08
Crystal size (mm)	0.13 × 0.11 × 0.05	0.16 × 0.15 × 0.05	0.24 × 0.15 × 0.12

Data collection			
Absorption correction	Multi-scan (*CrysAlis PRO*; Rigaku OD, 2019 ▸)	Multi-scan (*CrysAlis PRO*; Rigaku OD, 2019 ▸)	Gaussian (*CrysAlis PRO*; Rigaku OD, 2019 ▸)
*T*_min_, *T*_max_	0.587, 1.000	0.446, 1.000	0.022, 0.247
No. of measured, independent and observed [*I* > 2σ(*I*)] reflections	10937, 2522, 2427	13919, 2543, 2503	14718, 2436, 2433
*R*_int_	0.045	0.022	0.059
(sin θ/λ)_max_ (Å^−1^)	0.616	0.615	0.614

Refinement			
*R*[*F*^2^ > 2σ(*F*^2^)], *wR*(*F*^2^), *S*	0.042, 0.121, 1.06	0.022, 0.062, 1.07	0.036, 0.095, 1.11
No. of reflections	2522	2543	2436
No. of parameters	210	215	188
No. of restraints	8	9	5
Δρ_max_, Δρ_min_ (e Å^−3^)	0.87, −0.45	0.48, −0.38	1.75, −1.53

Computer programs: *CrysAlis PRO* (Rigaku OD, 2019 ▸), *SHELXT* (Sheldrick, 2015*a* ▸), *SHELXL2018* (Sheldrick, 2015*b* ▸), *Mercury* (Macrae *et al.*, 2020 ▸) and *SHELXL* in *WinGX* (Farrugia, 2012 ▸).

**Table 2:** Hydrogen-bond geometry (Å, °) for NaCTZ[Chem scheme1]

*D*—H⋯*A*	*D*—H	H⋯*A*	*D*⋯*A*	*D*—H⋯*A*
N3—H1N⋯O2*W*^i^	0.88 (1)	2.02 (1)	2.888 (3)	170 (4)
N3—H2N⋯N1^ii^	0.88 (1)	2.10 (1)	2.969 (3)	173 (4)
O1*W*—H1*W*⋯N2^iii^	0.87 (1)	1.91 (1)	2.778 (3)	171 (3)
O1*W*—H2*W*⋯O2^i^	0.87 (1)	2.29 (1)	3.148 (3)	169 (3)
O2*W*—H3*W*⋯O1^iv^	0.87 (1)	2.07 (2)	2.895 (3)	160 (4)
O2*W*—H4*W*⋯O3*W*	0.87 (1)	1.97 (2)	2.797 (6)	158 (4)
O2*W*—H4*W*⋯O3*W*^v^	0.87 (1)	2.20 (3)	2.915 (6)	139 (3)
O3*W*—H5*W*⋯O2^vi^	0.95	1.90	2.853 (5)	180
O3*W*—H6*W*⋯Cl1^vii^	0.92	2.76	3.683 (5)	180

Symmetry codes: (i) 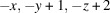; (ii) 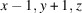; (iii) 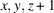; (iv) 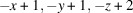; (v) 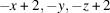; (vi) 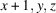; (vii) 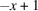, 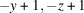.

**Table 3:** Hydrogen-bond geometry (Å, °) for KCTZ[Chem scheme1]

*D*—H⋯*A*	*D*—H	H⋯*A*	*D*⋯*A*	*D*—H⋯*A*
N3—H1N⋯Cl1	0.87 (1)	2.77 (2)	3.1942 (13)	112 (2)
N3—H1N⋯N1^i^	0.87 (1)	2.12 (1)	2.9337 (18)	157 (2)
N3—H2N⋯N2^ii^	0.87 (1)	2.23 (1)	3.0173 (18)	150 (2)
O1*W*—H1*W*⋯O1	0.87 (1)	2.29 (2)	3.0792 (16)	151 (2)
O1*W*—H2*W*⋯O2*W*^iii^	0.87 (1)	1.94 (1)	2.8075 (17)	176 (2)
O2*W*—H3*W*⋯N2^iv^	0.87 (1)	2.07 (1)	2.9308 (17)	171 (2)
O2*W*—H4*W*⋯O3*W*^v^	0.87 (1)	1.94 (1)	2.8053 (13)	173 (2)
O3*W*—H5*W*⋯O2^iii^	0.88 (1)	2.58 (3)	3.1279 (10)	121 (2)
O3*W*—H5*W*⋯N3^vi^	0.88 (1)	2.32 (2)	3.0506 (16)	140 (3)

Symmetry codes: (i) 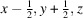; (ii) 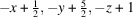; (iii) 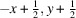, 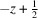; (iv) 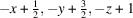; (v) 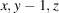; (vi) 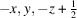.

**Table 4:** Hydrogen-bond geometry (Å, °) for CsCTZ[Chem scheme1]

*D*—H⋯*A*	*D*—H	H⋯*A*	*D*⋯*A*	*D*—H⋯*A*
N3—H1N⋯O1*W*^i^	0.88 (1)	2.00 (2)	2.864 (5)	168 (7)
N3—H2N⋯N1^ii^	0.88 (1)	2.25 (3)	3.048 (5)	151 (5)
O1*W*—H1*W*⋯N2^iii^	0.88 (1)	1.96 (3)	2.761 (5)	150 (6)
O1*W*—H2*W*⋯O3^iv^	0.88 (1)	2.05 (3)	2.867 (5)	154 (7)

Symmetry codes: (i) 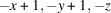; (ii) 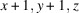; (iii) 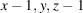; (iv) 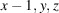.

**Table 5:** Selected bond lengths (Å) for polymorphic forms 1 and 2 of CTZ, and for salt forms containing CTZ anions

	S1—N1	N1—C1	C1—N2	N2—C2	S2—N3
CTZ, form 1	1.619	1.299	1.341	1.394	1.607
CTZ, form 2	1.620	1.309	1.344	1.391	1.590
NaCTZ	1.580 (2)	1.352 (4)	1.323 (4)	1.378 (3)	1.585 (2)
KCTZ	1.5979 (12)	1.333 (2)	1.323 (2)	1.3853 (19)	1.6086 (13)
CsCTZ	1.591 (4)	1.340 (5)	1.318 (6)	1.376 (5)	1.602 (3)
APUZOB A	1.598	1.335	1.333	1.379	1.618
APUZOB B	1.590	1.340	1.327	1.385	1.609
VEKBOF	1.577	1.333	1.306	1.385	1.599

**Table 6:** Bond lengths (Å) for coordination bonds in NaCTZ, KCTZ and CsCTZ

	NaCTZ	KCTZ	CsCTZ
*M*—N3			3.457 (3)
*M*—O1	2.360 (2)	2.7813 (11)	2.952 (3)
*M*—O2		2.7133 (11)	3.056 (3), 3.131 (3)
*M*—O3	2.365 (2)	2.6720 (11)	3.162 (3)
*M*—O4	2.464 (2)	2.7478 (11)	
*M*—Cl1		3.3257 (4)	3.7738 (9)
*M*—OH_2_	2.355 (2)	2.6269 (12)	3.244 (4)
	2.418 (2)	2.8493 (12)	
	2.438 (2)		

## References

[bb1] Aljohani, M., Pallipurath, A. R., McArdle, P. & Erxleben, A. (2017). *Cryst. Growth Des.* **17**, 5223–5232.

[bb2] Bernal, I. & Watkins, S. F. (2013). *Acta Cryst.* C**69**, 808–810. 10.1107/S0108270113015114 23907863

[bb3] Brydson, R. K. H. & Kennedy, A. R. (2024). *Acta Cryst.* E**80**, 806–810. 10.1107/S2056989024006078 PMC11223696 38974168

[bb4] Cametti, M., Nissinen, M., Dalla Cort, A., Rissanen, K. & Mandolini, L. (2006). *Inorg. Chem.* **45**, 6099–6101. 10.1021/ic060251u 16878909

[bb5] Farrugia, L. J. (2012). *J. Appl. Cryst.* **45**, 849–854.

[bb6] Groom, C. R., Bruno, I. J., Lightfoot, M. P. & Ward, S. C. (2016). *Acta Cryst.* B**72**, 171–179. 10.1107/S2052520616003954 PMC4822653 27048719

[bb7] Hankins, J., Lonsway, R. A., Hedrick, C. & Perdue, M. (2001). Editors. *Infusion Therapy in Clinical Practise*. Philadelphia: Saunders.

[bb8] Harlow, R. L. (1996). *J. Res. Natl Inst. Stand. Technol.* **101**, 327–339. 10.6028/jres.101.034 PMC4894611 27805169

[bb9] Johnston, A., Bardin, J., Johnston, B. F., Fernandes, P., Kennedy, A. R., Price, S. L. & Florence, A. J. (2011). *Cryst. Growth Des.* **11**, 405–413.

[bb10] Kennedy, A. R., Cruickshank, L., Maher, P. & McKinnon, Z. (2023). *Acta Cryst.* C**79**, 386–394. 10.1107/S2053229623007696 PMC10551880 37721716

[bb11] Leech, C. K., Fabbiani, F. P. A., Shankland, K., David, W. I. F. & Ibberson, R. M. (2008). *Acta Cryst.* B**64**, 101–107. 10.1107/S010876810705687X 18204216

[bb12] Macrae, C. F., Sovago, I., Cottrell, S. J., Galek, P. T. A., McCabe, P., Pidcock, E., Platings, M., Shields, G. P., Stevens, J. S., Towler, M. & Wood, P. A. (2020). *J. Appl. Cryst.* **53**, 226–235. 10.1107/S1600576719014092 PMC6998782 32047413

[bb13] Martins, V. M., Ziegelmann, P. K., Helal, L., Ferrari, F., Lucca, M. B., Fuchs, S. C. & Fuchs, F. D. (2022). *Syst. Rev.* **11**, 23. 10.1186/s13643-022-01890-y PMC8826711 35135630

[bb14] Mastropierro, P., Kennedy, A. R. & Hevia, E. (2022). *Chem. Commun.* **58**, 5292–5295. 10.1039/d2cc00979j PMC9040423 35403647

[bb15] Osterloh, F., Achim, C. & Holm, R. H. (2001). *Inorg. Chem.* **40**, 224–232. 10.1021/ic000617h 11170527

[bb16] Oswald, I. D. H., Lennie, A. R., Pulham, C. R. & Shankland, K. (2010). *ChemEngComm*, **12**, 2533–2540.

[bb17] Oyama, H., Miyamoto, T., Sekine, A., Nugrahani, I. & Uekusa, H. (2021). *Crystals*, **11**, 412.

[bb18] Paluch, K. J., Tajber, L., McCabe, T., O’Brien, J. E., Corrigan, O. I. & Healy, A. M. (2010). *Eur. J. Pharm. Sci.* **41**, 603–611. 10.1016/j.ejps.2010.08.013 20816757

[bb19] Paluch, K. J., Tajber, L., McCabe, T., O’Brien, J. E., Corrigan, O. I. & Healy, A. M. (2011). *Eur. J. Pharm. Sci.* **42**, 220–229. 10.1016/j.ejps.2010.11.012 21115114

[bb20] Raymond, K. N. & Girolami, G. S. (2023). *Acta Cryst.* C**79**, 445–455. 10.1107/S2053229623007088 PMC10625717 37610288

[bb21] Rigaku OD (2019). *CrysAlis PRO*. Rigaku Ltd, Yarnton, Oxfordshire, England.

[bb22] Rosokha, S. V., Lu, J., Rosokha, T. Y. & Kochi, J. K. (2009). *Phys. Chem. Chem. Phys.* **11**, 324–332. 10.1039/b811816g 19088988

[bb23] Seidel, R. W. (2018). *IUCrData*, **3**, x181324.

[bb24] Shankland, K., David, W. I. F. & Sivia, D. S. (1997). *J. Mater. Chem.* **7**, 569–572.

[bb25] Sheldrick, G. M. (2015*a*). *Acta Cryst.* A**71**, 3–8.

[bb26] Sheldrick, G. M. (2015*b*). *Acta Cryst.* C**71**, 3–8.

[bb27] Smith, G. (2013*a*). *Acta Cryst.* E**69**, m22–m23. 10.1107/S1600536812049562 PMC3588279 23476323

[bb28] Smith, G. (2013*b*). *Acta Cryst.* E**69**, m628. 10.1107/S1600536813029395 PMC3884976 24454151

[bb29] Smith, G. (2015). *Acta Cryst.* C**71**, 140–145. 10.1107/S205322961500056X 25652282

[bb30] Steuber, T. D., Janzen, K. M. & Howard, M. L. (2020). *Pharmacotherapy*, **40**, 924–935. 10.1002/phar.2440 32639593

[bb31] Teng, R., Wang, L., Chen, M., Fang, W., Gao, Z., Chai, Y., Zhao, P. & Bao, Y. (2020). *J. Mol. Struct.* **1217**, 128432.

[bb32] Yang, P., Wang, J., Jia, C., Yang, X.-J. & Wu, B. (2013). *Eur. J. Org. Chem.* **2013**, 3446–3454.

[bb33] Zaleskaya, M., Jagleniec, D., Karbarz, M., Dobrzycki, L. & Romański, J. (2020). *Inorg. Chem. Front.* **7**, 972–983.

